# Association of circulating histone H3 and high mobility group box 1 levels with postoperative prognostic indicators in intensive care unit patients: a single-center observational study

**DOI:** 10.20407/fmj.2022-008

**Published:** 2022-07-22

**Authors:** Ken Sawada, Yasuyo Shimomura, Daisuke Hasegawa, Tatsuhiko Harada, Tomoyuki Nakamura, Naohide Kuriyama, Yoshitaka Hara, Hidefumi Komura, Osamu Nishida

**Affiliations:** Department of Anesthesiology and Critical Care Medicine, Fujita Health University, School of Medicine, Toyoake, Aichi, Japan

**Keywords:** Damage associated molecular patterns, Histone H3, High mobility group box 1, Postoperative patient, Intensive care unit (ICU)

## Abstract

**Objectives:**

Damage associated molecular patterns (DAMPs) levels are associated with sepsis severity and prognosis. Histone and high mobility group box 1 (HMGB1) levels are also potential indicators of prognosis. We investigated the relationship between serum histone H3 and HMGB1 levels and the illness severity score and prognosis in postoperative patients.

**Methods:**

Postoperative serum histone H3 and HMGB1 levels in 39 intensive care unit (ICU) patients treated at our institution were measured. The correlation between peak histone H3 and HMGB1 levels in each patient and clinical data (age, sex, surgical time, length of ICU stay, and survival after ICU discharge), which also included the patients’ illness severity score, was examined.

**Results:**

Histone H3 but not HMGB1 levels were positively correlated with surgical time, the Sequential Organ Failure Assessment score, the Japanese Association for Acute Medicine acute phase disseminated intravascular coagulation diagnosis score, and the length of ICU stay. Both histone H3 and HMGB1 levels were negatively correlated with age. However, survival post-ICU discharge was not correlated with histone H3 or HMGB1 levels.

**Conclusions:**

Histone H3 levels are correlated with severity scores and the length of ICU stay. Serum histone H3 and HMGB1 levels are elevated postoperatively. These DAMPs, however, are not prognostic indicators in postoperative ICU patients.

## Introduction

Endogenous molecules released from cells are called damage-associated molecular patterns (DAMPs).^1^ DAMPs are also called alarmins because their release in connection with cell damage or cell death signals a crisis to neighboring cells.^2^

Among these molecules, histones and high mobility group box 1 (HMGB1) are increasingly attracting attention in intensive care settings as indicators of prognosis.^3,4^ Histones and HMGB1 are present in the cell nucleus. Histones are associated with DNA repair,^5^ while HMGB1 is associated with tissue repair^6^ and acts as a cytokine signal that promotes inflammation.^2^ Histones H2A, H2B, H3, and H4 form a complex with DNA called a nucleosome, which induces inflammation when released from the nucleus.^1^ HMGB1 has been reported to be associated with the occurrence of autoimmune diseases^7^ and systemic inflammatory response to infection including sepsis, which can cause coagulopathy, multiple organ failure, and death.^8^ DAMPs are released from cells even after tissue damage due to surgical trauma.^9–12^ In intensive care settings, both sepsis severity and prognosis were reported to be associated with increased serum histone H3 and HMGB1 levels.^3,4^ Serum HMGB1 and histone H3 levels that have been reportedly associated with an increased risk of death in sepsis are approximately 17 ng/mL^13^ and 9 ng/mL,^14^ respectively.

Studies using histones and HMGB1 as predictive markers of postoperative prognosis in various diseases have been previously reported.^9–13^ However, it is unclear whether DAMPs levels correlate with postoperative prognosis or symptom severity in postoperative patients requiring intensive care. In this study, we examined whether peak serum histone H3 and HMGB1 levels were associated with the illness severity score and prognosis (length of ICU stay and survival post-ICU discharge) in patients who were admitted to the ICU at our facility for postoperative management.

## Methods

### Patients

This single-center observational study was conducted between October and November, 2019. Thirty-nine postoperative patients admitted to the intensive care unit (ICU) at Fujita Health University Hospital were included.

Among the 39 patients who underwent surgery, 25 were scheduled and 14 were emergency surgeries. Among thoracic surgical cases, six were addressed using an open thoracic approach (five were performed by cardiovascular surgeons and one was performed by respiratory surgeons), and eight used a thoracoscopic approach (all performed by respiratory surgeons). Among abdominal surgical cases, ten were addressed using the open abdominal approach (seven were gastrointestinal procedures and two were transplant procedures), and six used the laparoscopic approach.

Residual blood samples from routine treatment-related blood tests (blood samples taken every morning during the postoperative ICU stay) were collected in accordance with a protocol approved by the Institutional Review Board and the Ethics Committee at Fujita Health University (approval number HM20-328). Anonymized, residual serum samples from patients were used to measure histone H3 and HMGB1 levels. Clinical data such as Sequential Organ Failure Assessment (SOFA), Acute Physiology and Chronic Health Evaluation II (APACHE II), and the Japanese Association for Acute Medicine (JAAM) acute phase disseminated intravascular coagulation (DIC) diagnosis (JAAM DIC) scores were also anonymized. The SOFA score is used to quantify the severity of sepsis on the basis of the degree of organ injury.^15^ JAAM DIC is the JAAM’s scoring system to diagnose DIC.^16^ The APACHE II score is the most widely used ICU mortality prediction score, and it includes up to 12 physiological and two disease-related variables collected within the first 24 h of ICU admission.^17^

### Measurement of histone H3 levels

Serum histone H3 levels were measured as previously described.^18^ Briefly, polystyrene microtiter plates (Nunc, Roskilde, Denmark) were coated with 100 μL of 1 mg/L anti-histone H3 antibodies (Shino-Test Corporation, Sagamihara, Japan). After washing and blocking, plates were incubated with 100 μL of diluted calibrator and serum samples for 24 h at 20–28°C. After washing, plates were incubated with 100 μL of anti-histone H3 peroxidase-conjugated antibodies (Shino-Test Corporation) for 2 h at 20–28°C. Thereafter, plates were re-washed and then incubated with the chromogenic substrate, 3,3,5,5-tetra-methylbenzidine (Dojindo Laboratories, Kumamoto, Japan). The reaction was terminated using 0.35 mol/L Na_2_SO_4_, and absorbance at 450 nm was measured using a microplate reader (Model 680, Bio-Rad, Hercules, CA, USA). A standard curve was created by measuring known levels of purified calf thymus histone H3 (Roche, Stockholm, Sweden). The lower detection limit of the enzyme-linked immunosorbent assay (ELISA) was 2 ng/mL, and linearity was observed up to 250 ng/mL.

### Measurement of HMGB1 levels

Serum HMGB1 levels were measured as recently described.^19^ Briefly, polystyrene microtiter plates (Nunc™: Thermo Scientific™, Tokyo, Japan) or black polystyrene microtiter plates (Corning^®^: Corning, One Riverfront Plaza, NY, USA) were coated with 100 μL anti-HMGB1 polyclonal antibodies (anti-peptide polyclonal antibodies, 3 mg/L) in phosphate-buffered saline (PBS). Plates were sealed with a thin adhesive-coated plastic sheet and incubated overnight at 37°C. Unbound antibodies were removed by washing each plate three times with PBS containing 0.05% Tween 20 (washing buffer), and the remaining binding sites in the wells were blocked by incubating the plates for 2 h with 400 μL/well of PBS containing 1% bovine serum albumin (BSA). After washing, 0.15 mol/L NaCl_2_ containing 1% BSA and 100 μL of each standard concentration and samples in 0.2 mol/L tris (pH 6.5) were added to the wells. Samples and purified human HMGB1 standards were diluted to 1:1.

Microtiter plates were incubated for 24 h at 20–28°C. After washing, anti-human HMGB1 peroxidase-conjugated monoclonal antibodies (100 μL/well) and plates were incubated at 20–28°C for 2 h, and 3,3,5,5-tetra-methylbenzidine (Dojindo Laboratories) or PS-atto (Luminogen, FUJIFILM Wako Pure Chemical Co., Osaka, Japan) was added to each well. The enzyme reaction was allowed to proceed for 30 min at 20–28°C. The chromogenic substrate reaction was halted using stop solution (0.35 mol/L Na_2_SO_4_), and absorbance was read at 450 nm. Luminescence after adding the chemiluminescence reagent (PS-atto) was measured using a luminescence microplate reader (Luminous CT-9000D, Dia-Iatron Co., Tokyo, Japan) after a 5-min incubation period.

### Statistical analyses

The highest measured levels of histone H3 and HMGB1 and the severity scores (SOFA, JAAM DIC, and APACHE II) for each patient during their ICU stay were considered. SOFA and JAAM DIC were scored on the same day as the highest histone H3 and HMGB1 values, respectively.^20^

The relationship between histone H3 or HMGB1 levels and severity scores and clinical data were determined through correlation analyses using Pearson’s correlation coefficient to assess normally distributed variables and Spearman’s rank correlation coefficient to evaluate non-normally distributed data. Serum histone H3 and HMGB1 levels from 90-day survivors were compared with those of non-survivors using the Mann–Whitney U test. All statistical analyses were performed using EZR statistical software (Saitama Medical Center, Jichi Medical University, Saitama, Japan),^21^ and p values <0.05 were considered statistically significant.

## Results

Clinical characteristics of 39 patients postoperatively admitted to the ICU at our institution between October 28 and November 8, 2019 are presented in Table 1.

Among the 39 patients, 25 were men and 14 were women. The median age of the included patients was 67 (range, 0–82) years. The median surgical time was 259 (range, 28–1142) min. The median length of ICU stay was 3 (range, 2–35) days.

The median SOFA, JAAM DIC, and APACHE II scores were 3 (range, 0–16), 1 (range, 0–8), and 13 (range, 2–27) points, respectively. At 28 days post-ICU discharge, the survival rate was 97.4% (one of 39 patients died). After 90 days, the survival rate was 90.9% (three patients died and six patients had unknown outcomes).

Median histone H3 and HMGB1 levels were 3.6 (range, 0–26.8) ng/mL and 7.5 (range, 1.5–23.8) ng/mL, respectively.

**Figure 1:**
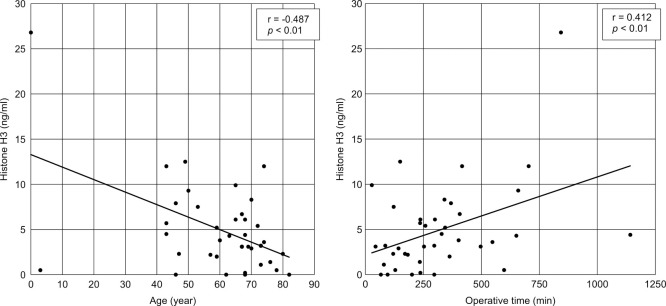
Correlation between histone H3 levels and age and surgical time Scatter plots of the histone H3 level versus patient information are shown. A significant negative correlation between the histone H3 level and age and a significant positive correlation between the histone H3 level and surgical time were revealed. Approximation lines were added to the graphs that showed a significant correlation.

**Figure 2:**
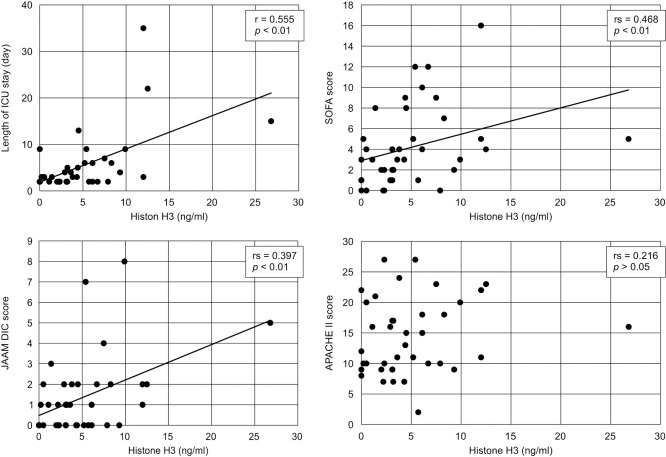
Correlation between the patients’ histone H3 level and the length of ICU stay and the patients’ illness severity score Scatter plots of histone H3 levels versus the length of ICU stay and severity scores are shown. Significant positive correlations between the histone H3 level and length of ICU stay, SOFA score, and JAAM DIC score are shown. No correlation between histone H3 level and APACHE II score is evident. Approximation lines were added to the graphs that showed a significant correlation. ICU, intensive care unit; SOFA, Sequential Organ Failure Assessment; JAAM, Japanese Association for Acute Medicine; DIC, disseminated intravascular coagulation; APACHE, Acute Physiology and Chronic Health Evaluation

Associations between histone H3 levels and clinical data are shown in Figures 1 and 2. Histone H3 levels and surgical time (r=0.412, p<0.01), length of ICU stay (r=0.555, p<0.01), SOFA score (rs=0.468, p<0.01), and JAAM DIC score (rs=0.397, p<0.01) showed a positive correlation. However, the APACHE II score showed no correlation, while age was negatively correlated with the histone H3 level (r=0.216, p>0.05 and r=–0.487, p<0.01, respectively). No correlation was found between sex and survival post-ICU discharge (data not shown).

**Figure 3:**
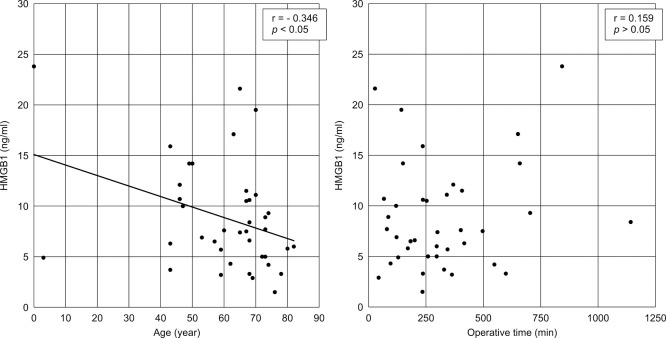
Correlation between HMGB1 levels and age and surgical time Scatter plots of the HMGB1 level versus patient information are shown. A significant negative correlation between the HMGB1 level and age is shown. There was no correlation between the HMGB1 level and surgical time. Approximation lines were added to the graphs that showed a significant correlation. HMGB1, histone and high mobility group box 1

**Figure 4:**
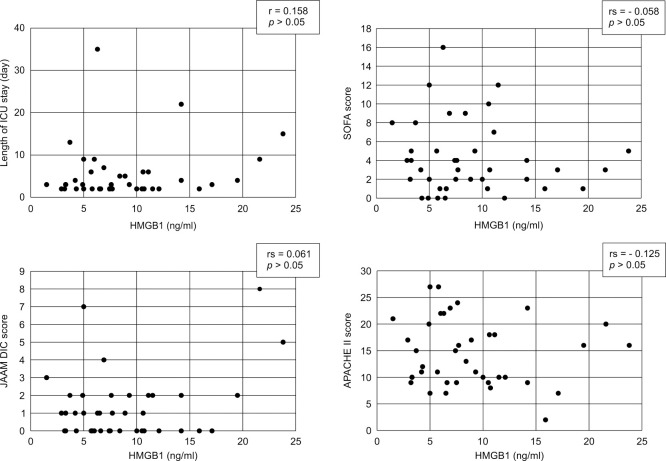
Correlation between HMGB1 and length of ICU stay and the patients’ illness severity score Scatter plots of HMGB1 versus the length of ICU stay and indicators of illness severity are shown. The HMGB1 level did not correlate with the length of ICU stay, SOFA score, JAAM DIC score, or APACHE II score. HMGB1, histone and high mobility group box 1; ICU, intensive care unit; SOFA, Sequential Organ Failure Assessment; JAAM, Japanese Association for Acute Medicine; DIC, disseminated intravascular coagulation; APACHE, Acute Physiology and Chronic Health Evaluation

Associations between the HMGB1 levels and clinical data are shown in Figures 3 and 4. No correlations between HMGB1 levels and surgical time (r=0.159, p>0.05), length of ICU stay (r=0.158, p>0.05), SOFA score (rs=−0.058, p>0.05), JAAM DIC score (rs=0.061, p>0.05), APACHE II score (rs=−0.125, p>0.05), sex and survival post-ICU discharge were identified. However, age was negatively correlated with the HMGB1 level (r=−0.346, p<0.05).

## Discussion

Associations between the highest histone H3 and HMGB1 levels and symptom severity and prognosis were assessed in postoperative patients admitted to the ICU. Although the results showed elevated postoperative serum histone H3 and HMGB1 levels in these patients, only histone H3 levels were correlated with severity scores and length of ICU stay, while no correlations were found between these DAMPs and illness severity scores and prognosis.

Histones and HMGB1 are usually not detected in healthy human blood.^22^ DAMPs released from cells and circulating throughout the body are highly cytotoxic and contribute to various organ disorders. Therefore, in intensive care, high serum histone and HMGB1 levels have been reported to be associated with sepsis severity and prognosis.^2,3^ Previous research in our lab revealed that extracellular histones can be found in the lungs and liver of an endotoxin shock mouse model via immunofluorescent staining.^23^ In this study, histone H3 and HMGB1 were detected in the serum of postoperative patients requiring intensive care. Furthermore, both serum histone H3 and HMGB1 levels were negatively correlated with age. This is likely because young people have healthier immune systems and much more muscle mass than older people, which allows higher serum DAMPs concentrations in younger compared with older people.

In this study, it was suggested that high serum histone H3 levels may reflect the degree of tissue and organ damage that occurred during surgery because it was associated with surgical time, length of ICU stay and multiple illness severity scores. However, high serum histone H3 levels were not associated with survival 28 or 90 days after discharge from the ICU. Therefore, increases in histone H3 levels may predict the short-term but not long-term prognosis.

High serum HMGB1 levels were not associated with surgical time, severity scores, length of ICU stay, or survival after discharge from the ICU. HMGB1, which is also called a death mediator,^24,25^ is elevated in cases of severe illness including sepsis. Therefore, our results may indicate that the condition of the postoperative patients in this study was not sufficiently severe to produce high enough HMGB1 levels to show an association with prognostic indicators.

Because neither high serum histone H3 levels nor high serum HMGB1 levels were related to the survival rate at 28 or 90 days after ICU discharge, the levels of these molecules may not correlate with ICU mortality prediction scores such as the APACHE II score.

Tissue injury causes DAMPs release from damaged cells, which induces an inflammatory reaction and tissue repair. However, high circulating DAMPs levels will cause sepsis and damage to remote organs. In patients who are capable of withstanding surgical treatment, circulating DAMPs levels may transiently increase. However, this could be a normal innate immunity response. The biological postoperative defense mechanism seems to be different from mechanisms that promote strong inflammatory and immune responses, such as sepsis.^26^

A limitation of this study is that it was performed at a single facility. Second, the number of patients enrolled was relatively small. Third, patients undergoing invasive procedures requiring postoperative management in the ICU were assessed without consideration of each patient’s underlying disease. Additionally, most patients were treated using endoscopic surgery. Therefore, findings after laparotomy or open chest surgery require further examination. It will also be useful to examine whether treated disease and the surgical methods that were used affect the results in the future.

In conclusion, our findings revealed that histone H3 and HMGB1 can be detected in postoperative patients’ serum. Furthermore, levels tended to be higher in younger compared with older patients. Additionally, histone H3 but not HMGB1 levels may be an indicator of illness severity and length of ICU stay in postoperative patients. However, elevated histone H3 and HMGB1 levels due to surgical invasion are not associated with the outcome, unlike prolonged and excessive immune responses such as sepsis.

## Figures and Tables

**Table1:** Clinical data

n=39	Median (range)
Age (years)	67 (0–82)
Gender (male:female)	25:14
Scheduled:Emergency	14:15
Operative time (min)	259 (28–1142)
Length of ICU stay (days)	3 (2–35)
SOFA score	3 (0–16)
The JAAM DIC score	1 (0–8)
APACHE II score	13 (2–27)
Histone H3 (ng/mL)	3.6 (0–26.8)
HMGB1 (ng/mL)	7.5 (1.5–23.8)

Thoracic surgery (open:thoracoscopic)	6:8
Abdominal surgery (open: laparoscopic )	10:6

Survival post ICU	Survivors	Mortality
28 days	38 (97.4%)	1
90 days (Unknown:6)	30 (90.9%)	3
